# A Phone Pal to overcome social isolation in patients with psychosis—Findings from a feasibility trial

**DOI:** 10.1371/journal.pdig.0000410

**Published:** 2024-01-12

**Authors:** Mariana Pinto da Costa, Kirat Virdi, Athanasia Kouroupa

**Affiliations:** 1 King’s College London, London, United Kingdom; 2 Queen Mary University of London, London, United Kingdom; 3 Essex Partnership NHS Foundation Trust, Essex, United Kingdom; 4 University College London, London, United Kingdom; Imperial College London Faculty of Medicine, UNITED KINGDOM

## Abstract

People with psychosis often experience social isolation due to stigma. Several volunteering programmes that exist in the community to support patients expect in-person meetings, requiring greater availability and commitment. This study investigated the acceptability and feasibility of remote volunteering over a smartphone for people with psychosis over 12 weeks, exploring its potential impact on both patients and volunteers. A total of 36 participants took part in the study. In the first phase, six participants were recruited in less than three weeks in London. All established contact with their match, and there were no study withdrawals. In the second phase, 30 additional participants were recruited in four weeks, across the United Kingdom. Most patients and volunteers reported that they primarily used audio calls to make contact, followed by text messages, WhatsApp messages and video calls. There were improvements in patients’ scores of quality of life, self-esteem, social contacts and symptoms, and in volunteers’ ratings of quality of life, physical activity, self-esteem, social comparison, and social distance towards people with mental illness. This study demonstrates that it is feasible, acceptable and safe to remotely connect volunteers and people with psychosis who are afar.

Trial registration: ISRCTN17586238 (registration date: 28/09/2018).

## Author summary

In this study, we aimed to explore the feasibility and acceptability of using remote volunteering over a smartphone to support people with psychosis, who are often socially isolated. We tested a 12-week intervention with 36 participants across the United Kingdom. In the first phase, six participants were recruited in less than three weeks in London, all established contact with their match, and there were no study withdrawals. In the second phase, 30 additional participants were recruited in four weeks, across the UK. Most patients and volunteers reported using audio calls to make contact, followed by text messages, WhatsApp messages, and video calls. Patients ratings improved for quality of life, self-esteem, social contacts, and symptoms, and volunteers ratings for quality of life, physical activity, self-esteem, social comparison, and social distance towards people with mental illness. This study shows that remote volunteering is feasible, acceptable, and safe to connect community volunteers and people with psychosis who are afar.

## Introduction

Volunteering might be a way to promote social relationships in patients with severe mental illness (SMI) and positive attitudes towards them from volunteers in the community [1]. The literature suggests that volunteering is linked with improvements in patients’ and volunteers’ physical and mental health [2]. However, existing volunteering programmes seem to be inflexible [3], not taking into consideration people’s preferences and the challenges of in-person meetings. Logistical problems such as long travel distances, busy schedules, additional commitments of volunteers or patients’ difficulties in leaving the house may all hinder face-to-face interaction. Although technology in mental health care is a priority in the National Health Service (NHS) of England Long Term plan to ease access to healthcare services and address health inequalities in different ways, the integration of modern technology into everyday life has been significantly overlooked [4].

Research findings report that over 80% of people with psychosis own a mobile phone which they use to remain digitally connected [5]. Technology may facilitate the way patients and volunteers establish and maintain a relationship via a volunteering programme, which in turn might benefit patients to be active members of their community, increase social cohesion and community participation. People with psychosis often fear and avoid social interaction [6]. Connecting them with a volunteer remotely via a smartphone may encourage them to use social skills, establish more secure attachments with others and become more physically active.

To explore this, the ‘Phone Pal’ intervention has been developed [7] guided by the Medical Research Council (MRC) framework for the development and evaluation of complex interventions [8] and the person-based approach [9]. The ‘Phone Pal’ intervention enables patients to use a smartphone provided by the research team to communicate for 12 weeks with a volunteer, using text, WhatsApp messages, e-mails, audio or video calls, thus enabling the participant to conduct informal conversation.

This study aims to evaluate the feasibility of the ‘Phone Pal’ intervention, to investigate its acceptability and participants’ response to the intervention. Performance of feasibility studies to assess outcome measures prior to larger trials is recommended to improve subsequent randomised controlled trial (RCT) data interpretation [10]. Research indicates that during intervention development, new outcome measures may need to be designed to align with the theoretical perspectives and hypothesised mechanisms of change reflected in the intervention. If researchers adopt an outcome measure in a RCT and the trial is not effective, the main problem may be the selection of an outcome measure that is insensitive to change or incongruent with the logic model of the intervention.

## Methods

### Design

A single centre, pre-post, single arm, mixed methods feasibility study was conducted in two phases. The first phase included a small sample of patients and volunteers recruited in London; the second phase incorporated a larger sample with volunteers recruited nationwide. The study obtained approval from the East of England–Cambridgeshire and Hertfordshire Research Ethics Committee (IRAS project ID: 244496). The study was registered in the International Standard RCT Number database (ISRCTN17586238). Full details have been published in the study protocol [11].

### Recruitment

A range of recruitment strategies were used to recruit people with psychosis followed in outpatient community mental health services, and community volunteers, as described in the study’s protocol [11]. Patient participants were eligible to participate if they: i) were 18 years or over; ii) had a clinical diagnosis of schizophrenia or a related psychotic disorder (ICD 10: F20-29); iii) were interested in having a volunteer with whom they would be in contact primarily through a smartphone for 12 weeks; iv) were receiving care in secondary NHS mental health services; v) had the capacity to provide informed consent; and vi) had sufficient command of English to complete the outcome measures. Volunteer participants were eligible if they: i) were 18 years or over; ii) were interested in having a patient with whom they would be in contact primarily through a smartphone for 12 weeks; iii) had capacity to provide informed consent; and iv) had sufficient command of English to complete the outcome measures. More information about the rationale for these inclusion criteria has been published elsewhere [7].

### Screening, consent and follow-up

Potential patient participants were referred to the study through the community mental health teams, clinical study officers, researchers or self-referral. Potential volunteer participants expressed their interest in the study directly to the main researcher via a phone call or e-mail. Individuals who met the inclusion criteria were invited to an in-person meeting with the researchers, during which the study information sheet was presented, consent obtained, and baseline outcome measures collected. Participants were provided with training, which covered an overview of the study, the intervention, the role and responsibilities of the volunteer, and guidance to engage and interact with their paired match, and access to support and supervision. All participants received a smartphone and training on how to use it. The participant was then enrolled in the study and paired with their match in a pragmatic way, matching the first patient with the first volunteer available throughout recruitment. At the end of the 12 weeks, participants were telephoned to arrange a follow-up interview [11]. The scales selected to assess these outcome changes are presented in Table 1.

**Table 1:** Outcomes and corresponding scales to assess them.

	Outcomes	Scales
**Patients**	Quality of life	Manchester Short Assessment of Quality of Life (MANSA) [12]
	Physical activity	International Physical Activity Questionnaire (IPAQ) Short-Form [13]
	Self-esteem	Self-Esteem Rating Scale Short-Form (SERS-SF) [14]
	Social comparison	Social Comparison Scale [15]
	Attachment	Revised Adult Attachment Scale–Close Relationships’ Version (RAAS) [16]
	Social contacts	7 days Social Contacts Assessment [17]
	Symptoms	Brief Psychiatric Rating Scale (BPRS) [18]
	Character of the relationship with the volunteer	Scale to Assess Therapeutic Relationship–Patients’ Version [19]
**Volunteers**	Quality of life	Manchester Short Assessment of Quality of Life (MANSA) [12]
	Physical activity	International Physical Activity Questionnaire (IPAQ) Short-Form [13]
	Self-esteem	Self-Esteem Rating Scale Short-Form (SERS-SF) [14]
	Social comparison	Social Comparison Scale [15]
	Attitudes towards people with mental illness	Social Distance Questionnaire [20]
	Character of the relationship with the patient	Scale to Assess Therapeutic Relationship–Volunteers’ Version [19]

### Data analysis

Descriptive analysis was conducted on an individual basis for participants who completed the baseline and follow-up outcome measures. This was done regardless of whether they completed the intervention or withdrew (‘intention to treat’ analysis). Outcome measures were assessed for completeness and the percentage of missing responses reported. To enable the calculation of the overall scales, individual mean imputation was performed, imputing the calculated mean for a participant to the responses to the other questions [21]. This statistical analysis was conducted using the Software Package for Social Sciences for Windows v. 24.0 (SPSS Inc. Chicago, IL).

Thematic analysis was used to investigate participants’ experiences with recruitment, access to training and support from the research team, and response to the intervention. To facilitate data coding and analysis, the imported data were processed using NVivo software version 11. The aim of the analysis was to identify key themes based on the perspectives of the participants. Following analysis of all transcripts by the main researcher (MPC), in the second stage, two researchers (AK and KV) were involved to ensure coding uniformity and validity. The research team then discussed and reviewed these themes to ensure their coherence, distinctness, and credibility.

## Results

### Recruitment and study retention

**Fig 1:**
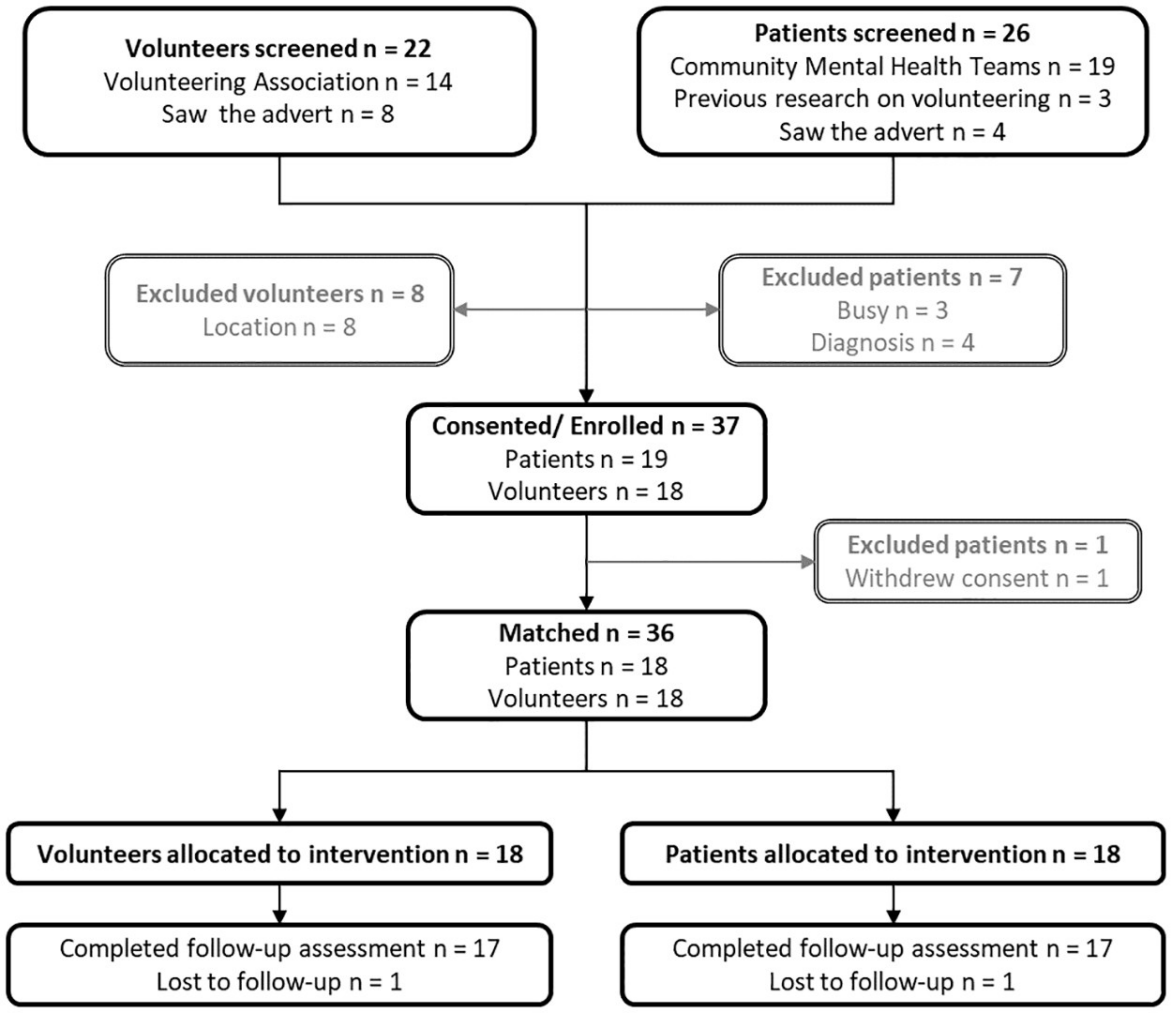
Study flow diagram.

The study recruitment process is outlined in the study flow diagram (Fig 1). For the first phase, recruitment of six participants took place in less than three weeks (23 October–9 November 2018). For the second phase, enrolment of the additional 30 participants occurred in four weeks (4 March 2019–29 March 2019). Recruitment to the pre-defined target of 36 (n = 3 patients and n = 3 volunteers in the first phase and n = 15 patients and n = 15 volunteers in the second phase) was achieved faster than anticipated (expected recruitment end date of 1 October 2019) due to the high interest from patients and volunteers to join the study. Several expressions of interest in being a volunteer were received from across the country (e.g., Cardiff, Oxford and Leeds) and internationally (e.g., Malta, Canada, Uganda and New Zealand). None of the participants expressed dissatisfaction with their match in the first two weeks. This article reports the baseline data from 19 patients and 18 volunteers, and the 12-week follow-up data from 18 patients and 17 volunteers (unless otherwise specified).

Patient participants heard about the study through their psychiatrist, clinical team, researchers or the adverts in the local NHS Trust. Volunteers found out about the study either via a presentation, from a volunteering association or from other volunteers, through social media or word of mouth (Table 2).

**Table 2:** Where participants heard about the study.

**Where patients heard about the study**	
**Community Mental Health Teams**	*“She was*, *the doctor*, *my mental health doctor was telling the coordinator that I need someone to talk*.*”* **(Patient 17)**
**Researcher**	*“I was in [the community mental health team]*, *waiting for a CPA [Care Programme Approach] and then I was approached by someone–a researcher*.*”* **(Patient 16)**
**Advert**	*“[Saw it in] the doctor’s surgery*, *just saw it on a noticeboard*.*”* **(Patient 3)**
**Where volunteers heard about the study**	
**Presentation**	*“I heard about this and I read also in the slide that there was the possibility to be part of the study*.*”* **(Volunteer 2)**
**Volunteering Association**	*“By other volunteers*, *so one of my friends had told me about this project*, *and I felt it was very interesting so… as I like a new challenge*. *I said ‘why not me*?*’ and I tried to contact the investigator and I was selected to be a volunteer*.*”* **(Volunteer 4)**
**Social Media**	*“I saw it on Twitter… Um*, *but then actually I saw it advertised around work in posters after I’d already emailed you about it anyway*.*”* **(Volunteer 17)**
**Researcher**	*“I heard this from the researcher… So direct word-of-mouth”*. **(Volunteer 12)**

### Reasons for taking part

Patients described different motivating and facilitating factors for choosing to take part (Table 3). Motivations included curiosity, i.e. having a new experience or interest in using technology, and usefulness, due to feeling mentally unwell, lonely or lacking friends, wanting to be occupied or interested in getting a smartphone. Patients also spoke about facilitators for their decision, such as convenience, e.g. it being a good time for them or not being a big burden.

**Table 3:** Patients’ motivations and facilitators to take part.

**Patients’ motivations**	
**Have a new experience**	*“My belief is that if you don’t know what it’s about you won’t know unless you give it a try*. *So yeah*, *I’ve given it a try now and it was brilliant*.*”* **(Patient 1)**
**Interest in the use of technology**	*“Just to monitor*, *monitor my own communications*, *and see how that goes*.*”* **(Patient 3)**
**Help with their mental health**	“*Well I decided to take part in the Phone Pal study because I think that*, *it might kind of help me a lot–especially when it comes to mental health*.” **(Patient 7)**
**Lack of friends and loneliness**	*“I’ve had that thought in me for ages that I wanna make more friends*. *‘Cos I’ve got one good friend*, *but… he’s not very well and he’s old… So I want not to be alone…if anything happened to him; I want to make sure I have more good friends*. *That’s why I wanted more friends*.*”* **(Patient 2)**
**Wanted to be occupied**	*“I just thought I’d try it out and see*, *to keep myself busy*. *And it turned out to be okay*.*”* **(Patient 3)**
**To feel better or normal**	“*Fight back the stigma of the mental health… and… go back to be … a normal person… And at the same time being able to cope up and go back to the community and enjoy the quality of life*.” **(Patient 7)**
**To have someone to speak with and trust on**	*“I thought it would be quite nice to have somebody that I could be honest about my schizophrenia to*, *because I obviously don’t talk about it to my housemates–they don’t know–and I don’t talk about it to my colleagues at work*, *they don’t know*. *And I obviously don’t talk about it to my students; it’s only my close friends that know*, *and then not even all my close friends know*.” **(Patient 6)**
**To get a smartphone**	“*The fact that they said that they would give me a phone at the end of it was*, *was definitely a good thing*. *So that was definitely a catch*.” **(Patient 4)**
**Facilitators for patients**	
**Being a good time for them**	*“I…personally thought it’d be an ideal time for myself to speak to others–you know*, *the volunteer–and to actually talk to them like someone that is a friend on the phone*.*”* **(Patient 1)**
**Not a big burden**	*“You could phone*, *you could text*, *you could do it at your own convenience; it wasn’t intrusive; you didn’t have to go out and meet them … or something like that …I would have had to budget time in for*, *and I’m very busy with my students and my preparation*, *and just my house-cleaning and my chores and my cat*. *So*, *it was easy to communicate with them because it was [over] a phone*.*”* **(Patient 6)**

Volunteers conveyed different factors that acted as motivations and facilitators (Table 4). These were to: ‘give’, their time, help others, contribute to patients’ social integration, address stigma against mental illness or support future interventions; and to ‘gain’, a new experience, an understanding of mental illness, a new smartphone based relationship, or for professional reasons, interest in technology or to get a smartphone.

**Table 4:** Volunteers’ motivations and facilitators to take part.

**Volunteers’ motivations**	
**‘Give’ their time to people**	*“Because I love doing volunteering job*, *I love giving … my time to people*, *people who need the help–I am very*, *very pleased to give help*.*”* **(Volunteer 3)**
**To help someone**	*“For some people maybe they just need a listening ear*, *phone calls*, *or they just need someone to kind of let off their steam to*, *either again*, *via phone calls or texts … But just*, *whoever*, *if they needed like a new friend*, *or if they just needed kind of like a bit of a breath away from whatever they were going…whatever was going on in their lives; I was just hoping to be that person*.*”* **(Volunteer 7)**
**Contribute to patients’ social integration**	*“I thought it would be nice to try and see whether this could be a way to… make people feel more connected*.*”* **(Volunteer 11)**
**Reduce the stigma against mental illness**	*“I chose to take part because I believe that mental health is an illness like any other*, *and I believe that the social stigma and isolation of individuals with things such as schizophrenia is absolutely atrocious*, *and that we should do whatever we can to encourage that*, *and if any research is going to open doors for individuals who are socially isolated… And so I’d like to think that by participating in the study I’m reducing the stigma around another mental illness*.*”* **(Volunteer 10)**
**Contribute to future interventions**	*“now with the fact that smartphones are everywhere*. *… it’s kind of the next stage*, *it’s like what possibly might be the future*.*”* **(Volunteer 1)**
**‘Gain’ a new experience**	*“The principal reason is because it’s my first experience as [a] volunteer and I wanted to try something different*.*”* **(Volunteer 2)**
**Understand more about people with mental illness**	*“it sounded like maybe it would open my mind a bit I guess to experience what people are like who have got either a mental illness*, *but probably get perceived in a certain way and their … reality it’s not as people would expect*. *So yeah*, *from a selfish point of view …to get an idea of what people are going through I guess and see what they’re like rather than just…coming up with some ideas or thinking what they might be like*, *but actually you don’t really know*.*”* **(Volunteer 5)**
**To establish a new relationship through a smartphone**	*“I just thought it would be… interesting to kind of see how and if it would be possible to build this kind of… friendship or relationship*, *with a stranger… just through a smartphone*.*”* **(Volunteer 11)**
**Interest in the use of technology**	*“Because I think these new technologies are a good tool for the future in every field*, *including medicine and including mental diseases*. *So*, *as I like new technologies*, *not being a technologist in this field*, *but I like it; I feel they are very good*.*”* **(Volunteer 4)**
**Professional development**	*“I thought that it’d be a good opportunity to volunteer*. *And I was curious*, *I was looking for volunteer opportunities to sort of beef up my CV*.*”* **(Volunteer 16)**
**To get a smartphone**	*“I just thought why not*, *and good advertising*, *and to get a smartphone at the end of it–yeah*.*”* **(Volunteer 7)**
**Facilitators for volunteers**	
**Not a big burden**	*“I think that it sounded like a good way to do it in terms of*, *it’s not all kind of necessarily a face-to-face thing to start with*, *so the technology helps kind of make that barrier to communication a lot easier; it’s less committing*.*”* **(Volunteer 5)**
**Easy to incorporate in everyday life**	*“This is such a simple thing that you could easily integrate into your life*.*”* **(Volunteer 18)**
**Good timing for them**	*“The timing is … perfect because I am having … more clients with complex issues really [that] I’m supporting so*.*”* **(Volunteer 1)**
**Frequent user of smartphones already**	*“I volunteered to do the study–’cos it made sense to me*, *’cos I’m always on my phone*.*”* **(Volunteer 10)**
**Safety of a phone**	*“I wasn’t worried about anything*. *And I think that comes from the safety of a phone…I think that’s the difference*. *Well perhaps if it was a face-to-face befriending and I was going to be meeting this person*, *perhaps I would have felt different*, *but I suppose there’s that boundary*, *with the fact that it’s through a phone … there’s a safety boundary*.*”* **(Volunteer 1)**

Convenience was a facilitator that encouraged volunteers to take part, i.e. the study not seeming to be a big burden, easy to incorporate in everyday life, happening at a good time for them or being a frequent user of smartphones already, perceiving them as safe.

### Initial expectations

Before starting the study, the initial expectations of patients included enthusiasm about meeting a new person, what their match would be like, making a friend, having someone to talk to, using a smartphone and expectation that they might become more active or that the volunteer would become their advocate.

Patients also expressed initial concerns about being linked with the volunteer, e.g. about what it would be like to communicate with a new person or worries about how the volunteer would be. Other concerns included finding it difficult to engage in a conversation, especially when unwell, whether it would be too much for them or for the volunteer, and being a burden for the volunteer. Additional issues related to the possibility of not getting along with their match. Nine patients said they did not have any immediate worries. Patients’ initial expectations are described in Table 5.

**Table 5:** Patients’ initial expectations.

**Patients’ initial enthusiasms**	
**Meet a new person**	*“It would be fun getting to know new people*.*”* **(Patient 16)**
**Ideas about their match**	*“Well I hoped they’d be something like what they have been*, *what they turned out to be like*.*”* **(Patient 2)**
**Make a friend**	*“I thought if I could make a friend*, *that would be great*.*”* **(Patient 18)**
**Having someone to talk to**	*“I thought I would have someone who I could chat to about things that worried me*, *or I wasn’t so happy about… I suppose generally about my problems–I just thought it would be nice to talk to somebody that was supportive about my mental health*.*”* **(Patient 6)**
**Using a smartphone**	*“Maybe using the smartphone’s gadgets and the WhatsApp and this and that*. *That seemed worth it*. *Just getting to know the new technology*, *and to work*, *to use it frequently*, *fluidly–fluently*. *And um*, *yeah*. *That’s it*. *So exciting*.*”* **(Patient 3)**
**Become more active**	“*Talking to people*, *finding new friends*, *and going out more and having this idea that it can tell me how I am*, *if I made my daily steps and my life*.” **(Patient 19)**
**The volunteer becomes their advocate**	“*I am expecting that my match or the volunteer will help me to become my voice*, *to fight the stigma of being a mental health patient*, *so that we can go back like a normal person in the community*, *wherein we can be able to express ourselves without having second thoughts or without*, *what to call it*, *without any inhibitions*.*”* **(Patient 7)**
**Patients’ initial concerns**	
**Communicating with a new person**	*“It would be kind of strange*, *but it turned out okay–it turned out to be quite normal*. *Er*, *just meeting new people and speaking to my volunteer*.*”* **(Patient 3)**
**Worried about how the volunteer would be**	*“That it could be dangerous because you’ve got somebody that you don’t’ know that has this stigma of mental health and you don’t know how they’re gonna behave*. *But in my case that wasn’t the case*, *so it was okay*.*”* **(Patient 4)**
**Difficult to engage in a conversation**	*“[in the] beginning I was worried about what am I gonna talk*. *What I am going to say*.*”* **(Patient 17)**
**Their mental state**	*“I was a little worried at first being mentally unwell*. *But …then after a little while… that just went away*.*”* **(Patient 1)**
**To be a burden for the volunteer**	*“I might offend her or*, *I don’t know … pestered her…”* **(Patient 19)**
**Not getting along with the match**	*“I thought we might not get on*. *But we… sparked–me and the volunteer we sparked a rapport straight-away*.*”* **(Patient 4)**

Prior to commencing the study, the enthusiasms of volunteers were varied and included an initial excitement at the thought of a new experience, communicating with a new person, using technology to connect with someone, helping a person or establishing a different relationship.

Four volunteers said they had no initial concerns; one justified this due to the safety of communicating through a phone. A volunteer worried that the patient would not engage. Additional volunteers’ concerns were rooted in themselves, with fears of not being able to support the patient, not knowing what to do if the patient was unwell, harming the patient and managing boundaries, such as the patient wanting to meet up. Other issues were based on patients’ behaviour towards them and on some occasions, being afraid of the patient. There were also worries regarding the interaction in that it could be awkward, or the matching could be unsuccessful. Volunteers’ initial expectations are described in Table 6.

**Table 6:** Volunteers’ initial expectations.

**Volunteers’ initial enthusiasms**	
**A new experience communicating with a new person**	*“I just…it was an entirely new experience; I just thought okay this should be interesting*. *Um*, *just kind of going forward and of course speaking to someone completely new*. *I was looking forward to kind of just*, *okay*, *seeing what it would be like*.*”* **(Volunteer 7)**
**Using technology to connect with someone**	*“One thing that was also exciting or curious … was interesting*, *[to] know a person*, *not in the traditional way that you meet in a bar or outside but with a phone*. *… It’s a different process of relationship that start with maybe a message then a call*, *then a video call and then you can meet*. *This was… a big expectation that then was realised*.*”* **(Volunteer 2)**
**To help a person**	*“I thought I could help this person to talk*.*”* **(Volunteer 3)**
**Establish a different relationship**	*“It’s nice to sort of make a connection with somebody that actually*, *that is a different relationship*. *You know … it’s come about differently*. *It’s like a lottery isn’t it*, *and it’s sort of…it’s like winning a ticket to go to a place that you’ve never been before*. *It’s interesting … because there’s so much limited information about that person*, *so it’s all really just kind of finding out new things about somebody and it…broadens your world really doesn’t it*?*”* **(Volunteer 1)**
**Volunteers’ initial concerns**	
**Patient would not engage**	*“Yes*, *I was worried that this person couldn’t*, *wouldn’t engage for example*.*”* **(Volunteer 3)**
**Being afraid of the patient**	“*I think I was apprehensive at the beginning*, *a bit worried…’cos obviously the stereotypes of people with mental health conditions*, *thinking that everyone’s cuckoo or everyone’s just a bit unstable*.*”* **(Volunteer 7)**
**Fear of not being able to support the patient**	*“In the beginning I*, *I didn’t know if I had the capacity to talk with him the correct way*, *if I will be positive for him*, *or not*. *But as time goes on I felt that that fear was not real*, *because he is a normal person with a problem*.*”* **(Volunteer 4)**
**Not know what to do if the patient was unwell**	*“I think I was a … tiny bit apprehensive um*, *of if there was anything that came up that I didn’t know how to manage*. *That’s the only thing; so just a bit worried that oh my gosh what if they disclose something that either scares me or worries me*, *or*, *what if they had really poorly-controlled mental health and they were experiencing kind of either symptoms or just going through a really bad time*, *that then would have made me…burdened me or bothered me myself*, *and not been able to manage it*.*”* **(Volunteer 7)**
**Fear of harming the patient**	*“I was apprehensive actually because I didn’t want to say that wrong thing*, *and I think that’s stayed on to the end; I don’t want to say something to make someone else feel bad*.” **(Volunteer 16)**
**Worries about crossing boundaries**	*“So at the beginning he sent me a selfie and then he sent me his address … and he was like asking me if I wanted to meet up*. *And I actually got a bit scared … I don’t remember what I told him; I think I … went off topic talking about other things*. *But … I didn’t feel comfortable*, *because I didn’t even know him and he was like suggesting to meet up–already*.*”* **(Volunteer 15)**
**That the interaction would be awkward**	*“I was maybe a little bit nervous because you know…I didn’t see this person in real life*, *and it was*, *yeah*, *just a little bit weird maybe*.*”* **(Volunteer 13)**
**Unsuccessful match**	*“Sometimes the … connection could not be very positive*, *people could … not match very well and instead of being kind of helpful could be…kind of a disturbance*.*”* **(Volunteer 4)**

### Sample

#### Socio-demographics

The majority of patients identified as male. In contrast, the majority of volunteers identified as female. None of the patients or volunteers selected non-binary gender, which featured amongst the options. The age of patients ranged from 21 to 57 years old, whereas the age of volunteers ranged from 20 to 67 years old (Table 7).

**Table 7:** Socio-demographics characteristics.

	Patients n (%)	Volunteers n (%)
**Age**		
mean (SD)	41.2 (10.8)	32.9 (14.8)
**Gender**		
female n (%)	6 (31.6)	12 (66.7)
male n (%)	13 (68.4)	6 (33.3)
**Country of Birth**		
England	13 (68.4)	6 (33.3)
Elsewhere	6 (31.6)	12 (66.7)
**Nationality**		
British	17 (89.5) a	9 (50.0) b
Other	3 (15.9)	12 (50.3)
**First Language**		
English	13 (68.4)	9 (50.0)
Other	6 (31.6)	9 (50.0)
**Ethnicity**		
White	4 (21.1)	14 (77.8)
Black	5 (26.3)	2 (11.1)
Asian	8 (42.1)	1 (5.6)
Mixed	1 (5.3)	0
Other	0	1 (5.6)
Prefer not to say	1 (5.3)	0
**Religious belief**		
No	3 (15.8)	7 (38.9)
Yes	16 (84.2)	11 (61.1)
Christianity	8 (50.0)	7 (63.6)
Islam	6 (37.5)	0
Hinduism	1 (6.3)	1 (9.1)
Other	1 (6.3)	3 (27.33)
**Marital status**		
Single/unmarried	14 (73.7)	13 (72.2)
Married/co-habiting/civil partnership	1 (5.3)	2 (11.1)
Separated/divorced	4 (21.1)	3 (16.7)
Widow/Widower	0	0
**Children**		
No	12 (63.2)	13 (72.2)
Yes	6 (31.6)	5 (27.8)
**Living situation**		
Live alone	14 (73.7)	3 (16.7)
With partner/family	2 (10.5)	8 (44.4)
With friends	0	2 (11.1)
In shared accommodation	3 (15.8)	5 (27.8)
Other	0	0
**Accommodation**		
Independent owned accommodation	2 (10.5)	5 (27.8)
Independent rented accommodation	8 (41.1)	12 (66.7)
Supported accommodation	8 (42.1)	1 (5.6)
Homeless/roofless	0	0
Other	1 (5.3)	0
**Years of education**		
mean (SD)	13.2 (4.7)	16.8 (3.3)
range	6–23	11–22
**Highest level of education achieved n (%)**		
Primary Education	0	0
Secondary Education	6 (31.6)	2 (11.7)
Tertiary Education	7 (36.8)	3 (16.7)
Higher Education	5 (26.3)	13 (72.2)
Other Education	1 (5.3)	0
**Employment status n (%)**		
Full time employment	0	6 (33.3)
Part time employment	2 (10.5)	4 (22.2)
Volunteering employment (non-paid)	2 (10.5)	0
Sheltered employment	0	0
Unemployed	10 (52.6)	1 (5.6)
Student	2 (10.5)	6 (33.3)
Housewife/husband	0	0
Retired	3 (15.8)	1 (5.6)
**Monthly income**		
mean (SD) GBP	898.2 (304.2)	1410 (1157.9)
range	500–1500	0–4500

^a^ one participant had dual citizenship

^b^ three participants had dual citizenship

The majority of patients were born in England (68.4%). They were predominantly British nationals (89.5%) and English reported being their first language (68.4%). Patients were predominantly of an Asian (42.1%) ethnicity. The majority (84.2%) reported having a religious belief (84.2%), of which most were Christians (50.0%), followed by Muslims (37.5%). In contrast, the majority of volunteers were born outside England (66.7%), had a non-British nationality (66.7%) and half did not have English as a first language. The majority were of White ethnicity (77.8%) and had a religious belief (61.1%), of which most were Christians (63.6%). Table 7 reports participants’ country of birth, nationality, first language, ethnicity and religion.

The majority of patients (73.7%) and volunteers (72.2%) were single, and most patients (63.2%) and volunteers (72.2%) did not have children. Whilst a high proportion of patients lived alone (73.7%) and in supported accommodation (42.1%), volunteers were living with a partner or family (44.4%) and in independent rented accommodation (66.7%).

The number of years of education in patients ranged from 6 to 23 years, with a mean of 13.2 years. The largest proportion (36.8%) had tertiary education; half were unemployed (52.6%). Patients received a monthly income of £500 to £1500. In contrast, volunteers had between 11 and 22 years as range of years of education, with a higher mean of 16.8 years. The majority had a higher education degree (52.2%), were employed (55.5%), and with an income ranging from £0 to £4500 (Table 7).

### Previous experiences

#### Mental health problems

The number of years since the patients had received their clinical diagnosis of psychosis varied from 1 to 27, with a mean of 13.3 years (SD: 8.6). Most patients (68.4%) had not been admitted to hospital in the past year. Only three volunteers had a previous history of mental health problems; all had received treatment, and two had required hospitalisation (Table 8).

**Table 8:** Personal experience of mental health problems*.

		n (%)
**Patients**	**Previous hospitalisations (past year)**	
	No	13 (68.4)
	Yes	6 (31.6)
**Volunteers**	**Lived experience of mental health problems**	
	No	15 (83.3)
	Yes	3 (16.7)
	**Received mental health treatment**	
	No	0
	Yes	3 (100)
	**Previous hospitalisations (ever)**	
	No	1 (33.3)
	Yes	2 (66.7)

*baseline data

### Volunteering

None of the patients had received support from a volunteer before.

*“No…I’ve been under the Mental Health Act for years*. *So I’ve had social workers and things like that*. *But I’ve never had a special volunteer before*.*”* **(Patient 2)**

Three volunteers reported having no previous volunteering experience; the remainder had volunteered previously.

*“Well I did…I volunteered abroad*. *I mean years ago… I volunteered for VSO*, *Voluntary Service Overseas–but that’s … different…it’s an adventure you go on in your 20s*.*”* **(Volunteer 1)**

*“No*. *It’s my first…experience yeah”*. **(Volunteer 2)**

*“Oh… in all kinds of settings; I’ve volunteered for a number of years with Samaritans*, *I’ve been a trustee of a number of organisations*, *a community centre*, *professional … I’ve had voluntary posts in professional associations that I’m a member of–as well as those say more community-based stuff*. *So*, *yeah*. *Probably lots more than even come to mind–I’d have to have a look at … my CV to remember how many things I’ve been roped into or volunteered*. *But yeah*,*…a pretty wide range of things*. *And I’ve worked in the voluntary sector for more than 30 years*.*”* **(Volunteer 18)**

### Smartphone use

Several patients interviewed said they had used a smartphone before, although one of them described difficulties in using it; some patients reported no previous experience in smartphone use.

*“I bought it new*, *but I didn’t know how to use the smartphone*, *so I just used it for…it’s got voice recognition*, *so I used to ask it things like…show me recent pictures of …and I might say an actor or something and it’s shown me a picture of the actor*. *Mostly I’d just use that*. *Because I didn’t know how to use the phone*.*”* **(Patient 2)**

*“No*. *I’ve got a normal house phone*. *Land*, *land-line*.*”* **(Patient 9)**

All volunteers stated that they had previously used a smartphone; one of them even described already owning two smartphones, one for work and the other for personal use.

*“Yes*, *I’ve got a personal one and a work one*.*”* **(Volunteer 1)**

At baseline, the majority (84.2%) of patient participants had owned a smartphone; only three had never owned one. All volunteer participants had used and owned a smartphone (Table 9).

**Table 9:** Usage of smartphones prior to the intervention*.

	Patients (n)	Volunteers (n)
**Have you used a smartphone before?**		
Yes, used or owned	16	18
No	3	0
**If yes, what did you mostly use the smartphone for?**		
Audio calls	13	18
Video calls	10	10
Text messages	14	16
Facebook messages	8	15
WhatsApp messages	9	18
E-mails	13	17
Other	7	9
None of the above	0	0
**If no, what would you like to use the smartphone for?**		
Audio calls	3	
Video calls	2	
Text messages	3	
Facebook messages	1	
WhatsApp messages	2	
E-mails	1	
Other	0	
None of the above	0	

*baseline data

### Usage of the intervention and the smartphone

The adherence to the intervention was primarily assessed by what participants reported at the end of the study in the 12-week follow-up qualitative interview.

#### Self-reported questions

There were some differences in how patients and volunteers reported their general use of the study smartphone. The majority of patients used it for audio calls, whereas most of the volunteers used it for text messages. More volunteers used WhatsApp messages than the patients, and an equivalent number of volunteers and patients used video calls and e-mails (Table 10). To contact each other, on occasions patients and volunteers reported using different methods of communication, although the numbers are similar. The majority of both patients and volunteers reported using audio calls to communicate with each other, followed by text and WhatsApp messages. (Table 10).

**Table 10:** Usage of smartphones as reported after the intervention*.

	Patients (n)	Volunteers (n)
**In general, what did you mostly use the smartphone for?**		
Audio calls	16	11
Video calls	1	1
Text messages	11	14
WhatsApp messages	7	10
E-mails	3	3
Other	0	1
None of the above	0	0
**To communicate with your match (volunteer/patient), what would you like to use the smartphone for?**		
Audio calls	15	12
Video calls	1	1
Text messages	12	11
WhatsApp messages	3	5
E-mails	2	0
Other	0	1
None of the above	0	0

*follow-up data (n = 17 patients and n = 17 volunteers)

#### Smartphone and apps

Although participants were told by the researcher that they could keep the smartphones after the study, six participants returned the smartphones at the end of the study follow-up meeting, stating that such a smartphone would be of no use to them as they did not work very well.

The app (mspy) was only purchased in the first phase of the study, not in the second phase. This was because the team faced issues with the app since it only worked if smartphones were connected to Wi-Fi, and when the app disconnected, it was no longer able to monitor communications. In addition, there were logistical issues, which caused difficulties in purchasing and installing the app in the smartphones provided before recruiting each participant. It was therefore not possible to collect the intended data.

For the step count app (accupedo) there were issues with recording the daily steps in the app. Some participants did not carry their phones with them all the time, and on other occasions data appeared not to have been recorded if the app were not opened every day. Another problem experienced was that data transferred from the smartphones to the study manager’s e-mail or WhatsApp were often poorly received. Hence, it was not possible to systematically obtain the individual daily step count data.

### Impact

#### Changes in outcome measures

The overall change in the outcome measures of patients and volunteers from baseline to follow-up are reported in Tables 11 and 12.

**Table 11:** Main patients’ outcome measures.

Patients’ outcome measures	Baseline mean (SD)	Follow-up mean (SD)
**Quality of life**	4.3 (0.9)	4.4 (0.9)
**Self-esteem**	14.8 (25.6)	17.2 (21.8)
**Adult attachment**:		
**Close**	3.2 (1.0)	3.3 (0.8)
**Dependent**	2.9 (0.9)	3.1 (0.8)
**Anxiety**	2.7 (1.0)	2.8 (0.8)
**Social comparison**	65.3 (18.9)	64.9 (18.5)
**Social contacts**	3.2 (1.9)	3.6 (2.2)
**Symptoms**	44.6 (10.2)	43.6 (15.3)
**Physical activity**	2442.5 (4145.1)	1272.2 (1583.5)

**Table 12:** Main volunteers’ outcome measures.

Volunteers’ outcome measures	Baseline mean (SD)	Follow-up mean (SD)
**Quality of life**	5.5 (0.7)	5.6 (0.6)
**Self-esteem**	33.6 (14.5)	35.4 (14.6)
**Social comparison**	69.2 (11.6)	73.8 (12.0)
**Social distance**	11.4 (3.9)	11.0 (5.1)
**Physical activity**	2905.3 (2470.7)	3914.4 (3315.9)

For patients, overall scores tended to increase from baseline to follow-up in their assessments of quality of life, self-esteem, social contacts, close interpersonal relationships, symptoms, as well as for attachment, whereas the overall values of social comparison and physical activity decreased.

In terms of patient symptomatology, an improvement in scores from baseline to follow-up was observed. This was owing to improvements in the manic, negative and positive symptom sub-scales; the values of the depression sub-scale increased at follow-up (Table 13).

**Table 13:** Patient symptoms.

BPRS	Baseline mean (SD)	Follow-up mean (SD)
**Depression**	9.4 (4.6)	10.3 (5.9)
**Manic**	7.4 (2.3)	6.9 (1.9)
**Negative**	7.2 (2.5)	6.3 (2.2)
**Positive**	10.8 (5.2)	9.9 (4.8)
**Total**	44.6 (10.2)	43.6 (15.3)

For volunteers, overall, the measures’ values tended to improve from baseline to follow-up in all outcome measures, i.e. quality of life, self-esteem, social comparison, social distance and physical activity.

### Positive impact

Patients described different areas in which the communication with the volunteer was helpful. This positive impact varied from meeting a new person, learning how to make friends, being occupied, heard and supported and getting advice from the volunteer. Some mentioned these interactions made them feel less alone, cared for, close to someone afar or more confident (Table 14).

**Table 14:** Positive impact for patients.

Helpful for the patients	
**To meet a new person**	*“Just in getting to know more people*.. *through the [Phone Pal] … which bridged a gap of introduction …which was helpful*.*”* **(Patient 3)**
**Learned how to make friends**	*“It’s taught me that I can make friends*. *Even if you don’t really know the person…you could just get on the phone like what we got on the phone*: *make friends over the phone*.*”* **(Patient 2)**
**To be more occupied**	*“Sometimes just sitting watching the telly*, *bored stiff and you don’t know what to do with yourself*, *and then the phone will ring*, *hello*, *and it’s somebody*. *‘Hello is it [name]*, *yeah*?*’ and then…that’s it…it’s almost like a friend waiting…to light up and I can talk*.*”* **(Patient 1)**
**To feel cared for**	*“It was nice to see that somebody was calling me*, *interested to have a chat with me–even though I couldn’t respond*. *That was quite nice*, *you felt like somebody cares*.*”* **(Patient 18)**
**To get advice from the volunteer**	“*[The volunteer] helped again*, *she advised me about the carer team*, *medicine*, *yeah*.”**(Patient 17)**
**To be heard**	*“It was nice talking to them and they were understanding about my thoughts and about my life*.*”* **(Patient 5)**
**To feel less alone**	*“That I’m not the only person going through what I’m going through*.*”* **(Patient 4)**
**Felt close to someone afar**	*“The lady [in Oxford]… when we talk*, *it’s just like this*, *the person in front of you*.*”* **(Patient 17)**
**To be more confident**	*“I’m glad the experience*, *it’s taught me a lot*: *it’s taught me to be confident; it’s taught me I don’t have to be scared; if anything happens to [my only friend]*, *I can make friends again–it’s quite easy*, *just speak to them over the phone*, *get to know them and meet up with them*. *And then it goes alright*.*”* **(Patient 2)**

Volunteers also described different areas in which the communication with the volunteer had made a positive impact on them, e.g. meeting a new person, making a connection, feeling comfortable with the patient and encouraged by them, and finding common ground between them. Several volunteers found it helpful to understand more about mental health and reported changing their attitudes towards people with mental illness, asserting that it helped them to put their own problems in perspective. Volunteers also stated that it was useful to feel closer to someone afar and that it felt good to be able to help someone, to contribute to a new research study and to get to know someone over a smartphone (Table 15).

**Table 15:** Positive impact for volunteers.

Helpful for the volunteers	
**To meet a new person**	*“I gave a history of a person that is important*. *A new person that I know…I cannot say a ‘friend’ because it’s too early but a new person that I’m in contact with that is something deeply important in the life of a person–stay in contact with the other person also is …something very important that I get*.*”* **(Volunteer 2)**
**Connect with someone**	“*It felt positive when they replied*. *It was nice to get that response and feel like oh we’re… progressing in the getting to know each other stage and … it was nice*, *it was getting to know someone new*.*”* **(Volunteer 14)**
**Felt comfortable with the patient**	*“Like I just feel comfortable and at ease*. *So I don’t feel…like she could pose any risk or any threat to me in any way*, *and I don’t feel uneasy about meeting her*, *or like it’s unsafe or like she’s a stranger*.*”* **(Volunteer 7)**
**Felt encouraged by the patient**	*“He was like a… caring person*, *like he would also remember details about my life*. *He remembered where I was from*, *like tried to say a few words in Lithuanian as well*.*”* **(Volunteer 15)**
**To find commonalities**	*“It was quite nice to hear that we had things in common*, *you know*? *Kind of coincidentally and we kind of had a little bit…like you could tell by … what he’s passionate about and stuff*, *and that was interesting to me to… It’s like if you…bump into someone on the bus and you end up having a really nice conversation that’s a surprise sort of thing–it’s kind of that feeling really*.*”* **(Volunteer 1)**
**Understand more about mental illness**	*“It was helpful for me because gives me … a new perspective of what is a mental patient*, *what we…people not suffering from these kind of disease can do to help these patients*. *To break a little the discrimination they suffer*.*”* **(Volunteer 4)**
**To change attitudes towards mental illness**	*“That this is a very good experience*, *an experience that can change your way to see other people and to understand how … I saw people with mental health problems are not so different from*, *us*, *with all the difficulties*, *with all the problems that appear but…are normal people that you can hang out or include and talk with*.*”* **(Volunteer 2)**
**To put their own problems into perspective**	*“He made me realise that the problems that I have are just simple and … I shouldn’t be thinking too much about it*, *because there are things that could go really wrong*, *and these are the kind of things that you have to be grateful about*.*”* **(Volunteer 12)**
**Felt closer to someone afar**	“*It gives me that impression that the world is very small*. *So we can be together anywhere in the world*. *I travel frequently and I talk with other persons and the idea I have when I talk with other persons*, *it was the same that I have when I talk with my match; it brings people together*. *We can be very near each other even thousand kilometres away*, *and if we are together we can help him–because it’s the mind*.. *that needs help*. *It’s not the body …it’s to talk*, *to give support*.” **(Volunteer 4)**
**Felt good to be able to support someone**	“*I really like to help people*, *so I think…it felt good to do something good… interacting with him and when he told me that he would like to … still keep in touch with me I think that I was helping him*, *like to get from his daily routine or something like that*. *So it really made me feel good*.” **(Volunteer 9)**
**To feel you are contributing to an interesting research study**	*“It was fun because as I used the smartphone so much*, *I used the same tool to try to help someone*. *I was … convinced that I was helping the future of mental health and that for me–as I am a very curious*, *and I have a open mind*, *and adventure mind–it was also very good for me to see how can I contribute to improve the use of this tool*.*”* **(Volunteer 4)**
**Interesting to get to know someone over a smartphone**	*“I think that it was an interesting experience and nice to get to know someone… in a way that’s quite different to how I normally would*, *so over the phone rather than small talk in person*.*”* **(Volunteer 17)**

### Challenges

Most patients responded that there was nothing unhelpful to them about being in contact with the volunteer over the smartphone. There was a patient who described feeling they were let down. Others were sorry for not having met the volunteer, for not having spoken more or realised that they had not paid as much attention as they would have liked.

*“Um*, *not disappointed*, *but like maybe a bit let down*, *I think*. *like she was volunteering and maybe she could have made just like a little bit more effort to talk to me sometimes*.*”* **(Patient 16)**

*“I feel sad that I cannot get to meet the volunteer–that [I] speak with*. *Yeah*, *I feel sorry about that*.*”* **(Patient 8)**

*“I just didn’t pay as much attention to it as I should have*.*”* **(Patient 10)**

Similarly, most volunteers did not label anything as unhelpful, although one of the volunteers shared their disappointment at not seeing results. A few volunteers said that they lost interest in their match, felt guilty when they were not speaking to the patient, that it required a lot of effort to talk to the patient, or worried if they had done something wrong.

*“I was disappointed*. *Because I think I was very excited*, *very willing in the beginning and then when he started to not communicate with me anymore*, *I had doubts about myself*, *thinking*, *what did I do wrong*, *did I say anything wrong… What I did*. *I did what*, *everything I could do*, *but without results–I couldn’t see results*.*”* **(Volunteer 3)**

*“The amount of effort necessary that was required to contact her*. *I felt like she didn’t necessarily feel as invested in it as I was*.*”* **(Volunteer 10)**

*“I personally you know*, *wanted to make this like a friendship that’s positive; I think I should have managed my expectations as well to begin with*, *rather than expecting a positive outcome–which is what I do in most things anyway; in my life I just expect a positive outcome*. *But maybe I should have fully considered the other aspect of that*.*”* **(Volunteer 16)**

*“Guilty when I don’t send text messages every day because I feel like it’s such a little thing to do*. *When I used to forget the phone I’d be like ‘oh*, *how could you*? *Like what if they’ve sent a message and haven’t replied*?*’*. *Thankfully that was never the case*, *but it was sort of a sense of responsibility in terms of ‘you should be there if they need you*, *or if they’ve sent you a text message you should reply quickly to show them that you are there if they want to talk’*.*”* **(Volunteer 14)**

“*My only concern is that… I’ve done something*, *inadvertently done something that I shouldn’t have done*.” **(Volunteer 18)**

### Risk assessment and adverse events

In the second phase of the study, three patient participants reported adverse events either during the intervention or at follow-up. These consisted of the loss of study equipment, the incorrect use of study equipment and an external, unrelated and unexpected event which occurred during the time of the study. These were discussed within the Phone Pal team, and where deemed necessary, the clinical team were alerted. There were no Serious Adverse Events.

### Access to support and supervision

The lead researcher was able to contact all participants monthly throughout the study. Participants shared their experiences with access to support and supervision at the end of study interviews.

In the first phase of the study, the three patients were pleased with the information and training received at the beginning and throughout the study, and one described a feeling of safety. In the second phase of the study, whilst all the patients were satisfied with the support received, one suggested the option of fortnightly contact with the study coordinator, for those who may prefer it. The first three volunteers were pleased with the initial training and access to support throughout. In the second phase of the study, one volunteer suggested that videos with case studies would be potentially helpful (Table 16).

**Table 16:** Participants’ views on training and support.

**Patients’ views on the initial training**	
**Pleased with the training**	*“You telling me exactly what’s gonna happen*, *and then [my match] coming on the phone and then it being like that*. *And then keeping checks on everything*. *And I like that*, *I like that–that was good; it made me more confident*. *We know exactly what was gonna happen*. *Exactly the rules*. *We went through the forms pretty good–it’s a long form but have done it pretty good*.*”* **(Patient 2)**
**Patients’ views on access to support and supervision throughout the study**		
**Pleased with the support**	*“Yeah*, *very*. *[Support] very… very fulfilling*, *very*, *very helpful*.*”* **(Patient 3)**
**Possibility of more frequent support**	*“Maybe once every two weeks maybe*. *Or …no*, *once a week is too much*. *… But then some people might not want that*, *so… maybe if we kind of think about people’s preferences*, *I think so you can ask them at the interview*, *or whenever*, *if*, *like they can tick a box or something*. *Be like once a month or twice a month or if some people want more than that*.*”* **(Patient 10)**
**Volunteers’ views on the initial training**		
**Pleased with the training**	*“The briefing that you gave me beforehand–which was really useful*. *I’d really*, *really highly recommend …that presentation that you gave us on communication skills–really good*, *really useful*. *I sat with most of my calls I had it in front of me*.*”* **(Volunteer 10)**
**Suggested videos with case studies**	*“I think that maybe having a video or some previous information on*, *like more case studies would have helped*. *‘Cos I had seen like here’s how it happened*, *here’s what*, *this is happening*, *and here’s what your role would be and this is how it will happen–hopefully*. *And all of that*.*”* **(Volunteer 16)**
**Volunteers’ views on access to support and supervision throughout the study**		
**Pleased with the support**	*“I think I got the support I needed*.*”* **(Volunteer 15)**	

## Discussion

### Strengths and limitations

A strength of this study was that the protocol was followed systematically. Recruitment and retention rates were high, as was data completeness, reinforcing the suitability of the study procedures adopted. The ability to recruit patients from NHS settings and volunteers from the community is encouraging for external validity, generalisation and applicability of the findings to a future RCT trial [22]. In fact, in the second phase of the study, recruitment of volunteers was nationwide, confirming people were interested in sharing their time and providing support, even across large distances. The study also had some limitations. Firstly, as in other psychosocial interventions, the researchers had little control on how the intervention was delivered, relying on participants’ reported outcome measures and interviews. Secondly, the technology used within this study to monitor and confirm behavioural outcome changes, i.e., the two apps failed to work as expected, and so the exact adherence to the intervention is dependent on follow-up self-reported data and qualitative interviews. A third issue is use of only two discrete temporal assessments, at the beginning and end of the study. Only one follow-up limits understanding of how participants’ outcome measures may change with time and whether those changes that are sustained over time. Future research should include additional time points for follow-ups.

### Comparison with the literature

#### Feasibility of recruitment, retention and overall study procedures

Recruitment to the Phone Pal study was feasible and the rate higher than similar studies on digital mental health interventions [23]. This may be related to the use of multiple recruitment strategies, the short duration, flexibility and appeal of the intervention to potential participants, as well as the offer of smartphone provision as an attractive incentive.

In the Phone Pal study, it was feasible to recruit participants following the established eligibility criteria. A previous survey reported that the majority of patients preferred a volunteer who had personal experience of mental illness [24]. However, this trial did not provide this. According to the volunteers’ self-reported information only three volunteers described previous experience of mental illness. The fact that the age of the volunteers recruited in the Phone Pal study, although young, were slightly higher than other averages, could also offer an explanation as to why none of the volunteers withdrew consent. Previous studies support the notion that older people are more likely to commit to volunteering [1]. In terms of the employment status of the volunteers, the Phone Pal study contrasts with reported variations of the employment profile in the literature, i.e. either employed, unemployed, students or retired [25]. The fact that most volunteers were employed may be associated with their perception that study participation was not a burden, and easy to incorporate in their everyday life. Indeed, some volunteers reported that face-to-face meetings would be more burdensome and inconvenient.

No participants requested to be re-matched in the initial 2-week period. This suggests that participants were satisfied with their matches. However, it may raise the question as to whether the envisioned threshold is too short for two people to determine whether they are compatible.

Both baseline and follow-up assessments were conducted face-to-face. The baseline assessment required an in-person meeting to offer participants the study smartphone. A face-to-face follow-up assessment was conducted for two reasons. Firstly, this was the intervention’s first testing, and therefore potentially benefitted from a one-to-one in person study closure with a researcher. Secondly, the end of study assessment covered the questionnaire measurements followed by the qualitative interview. This would be potentially too long to be performed as one continuous remote assessment.

The CONSORT 2010 statement extension for randomised pilot and feasibility trials, provides a checklist to guide the reporting of data collection measures. For example, there is a reference to a feasibility study where the proportion of the acceptable missing data was established to be less than 10% [26]; this study performed much better than this.

In relation to the smartphone data usage, the problems experienced with the two smartphone apps are similar to those previously described in mental health literature, highlighting the potential technical challenges of reliable sensor data collection from mobile platform devices, e.g. the necessity of active user engagement as with the step count app [27]. With respect to the step count app data, former research has pointed out similar issues to those experienced in this study, suggesting caution in their use for monitoring physical activity [28]. Future research could use other ecological momentary assessment (EMA) tools, to capture information about participants’ behaviour in real time, and be able to establish effectively the intervention adherence and fidelity.

Given this was a feasibility study, there were no specific trial progression criteria aside from the time for ending recruitment. This was detailed in order to further understand the enrolment barriers and facilitators, which would later influence progression criteria definitions for the future trial. Commonly, progression criteria can range and encompass figures of recruitment, retention, programme implementation, achieved measures, fidelity, factors affecting protocol adherence and acceptability [26].

Only one patient participant withdrew consent from intervention participation, however, they still agreed to attend the study follow-up assessment. No volunteers dropped out of the intervention. There was an overall high retention rate for both patients (94.7%) and volunteers (94.4%), with only one patient and one volunteer lost to follow-up. These encouraging findings may be explained by the flexible nature of the intervention, use of remote communication, short study duration and small sample size.

Other studies reported unplanned absences from volunteers and higher levels of volunteer attrition [29]. The importance of the self-regulation between volunteers and the organisation in the decision to drop out or persevere has been previously recognised [30].

The varying strategies adopted by the research team may have contributed to higher retention rates than those published in other studies of digital interventions. It has been suggested that less than 5% loss to follow-up may lead to an unimportant level of bias, whilst 20% or greater loss to follow-up poses a substantial threat to a trial’s internal validity [31]. A mind-set of regular communication, positivity and study ownership was perhaps crucial for the Phone Pal research team, as well as for the participants.

#### Usage of the intervention

In the Phone Pal study, 100% of patients who started the intervention, communicated at some point with their volunteer. This is an encouraging finding and different from that observed in an RCT testing a face-to-face befriending programme where 22% of the patients never met the volunteer, a study which reported numerous problems in the implementation of the programme as initially envisioned [3].

Both patients and volunteers reported that they contacted each other mostly through audio calls, followed by text messages. This is in line with the emerging data on how people with schizophrenia engage with digital technology [32]. In a previous survey, patients with psychosis ranked their preferred digital communication methods in the order of text messages, WhatsApp messages, e-mails and Skype [24]. Of note is that although audio calls appeared as least preferred, this was because it was not amongst the listed survey options of ‘digital methods’ since audio calls were viewed to be a less novel and a somewhat established communication method. Still, some of the patient participants proactively named them, adding them to the possible list of communication methods [24].

#### Acceptability of, and response to, the intervention

These results have demonstrated that communication over a smartphone between a patient and a volunteer is acceptable. These findings might also suggest that such interactions may be more acceptable than regular face-to-face support given the recruitment rates, the minimum rates of loss to follow-up and the participants’ feedback via qualitative interviews at the end of study [33].

The number of AE in this study was minimal. A recent review about reporting of AE in RCTs has noted that the data collection, reporting and analysis of AE in clinical trials is inconsistent and has emphasised that RCTs as a source of safety are underused [34].

Although the outcome changes concern a small sample size with no control group, patients demonstrated a tendency towards improvement in their ratings of quality of life, self-esteem, social contacts and symptoms, and volunteers reported a tendency towards an improvement in their scores of quality of life, self-esteem, social comparison, physical activity and social distancing.

One possible explanation for the decline in patients’ values of physical activity could be that, once connected remotely with a volunteer, patients felt more engaged digitally and were therefore less inclined to be physically active. However, there was one patient who had an extreme value at baseline; this outlying data was absent at follow-up. Additionally, the social perception of patients worsened during the study. It is possible that by comparing themselves with their volunteer, they may have thought less of themselves by the end of the study period. It has been documented that social media can have an adverse impact on social comparison; this intervention might have had a similar effect [35].

In the Phone Pal study, a range of observer rated (i.e. BPRS) and self-reported outcomes were utilised, some of them concerning behavioural outcomes (e.g. social contacts and physical activity). Previous literature has described step count as a self-regulation technique, with some suggesting smartphones as an increasingly useful tool to promote physical activity [36]. A recent review of smartphone use, however, identified their main impact to be a reduction in physical activity, although it also pointed out a potential for positive use and physical activity encouragement [37]. A previous randomised trial of a smartphone based platform used for several interventions aimed at increasing physical activity used the smartphone to record the daily step count, the primary outcome. However participant attrition in this fully digital research study was high; only 17% of the 2783 participants completed all four planned interventions [38]. This is a higher attrition than that reported in a systematic review on interventional digital health trials that influence physical activity [39].

Importantly, an increase in social contacts might not translate into quality of the contacts or meaningful relationships since some contacts might be negative and prejudicial [40]. In the Phone Pal study indicators of social contacts were assessed with more objective quantifiable variables, such as the number of social contacts [17] and living alone. However, loneliness, the subjective emotional appraisal of the extent and quality of social relationships, was not assessed quantitatively. Still, satisfaction with social relationships was assessed through the sub-scale of quality of life, which is a subjective measure.

In patients’ attachment assessments, all values were slightly higher at follow-up, i.e. closeness, dependency and anxiety. High scores of anxiety represent worry about not being liked, high scores of dependency suggest being reliant on others, and a maximal score for closeness signifies the perception of being in proximity to others as easy [41].

The findings from the Phone Pal study also suggest a potential beneficial impact that volunteering may have on volunteers. These observed benefits are in line with published literature on the positive effects of volunteering. A narrative synthesis [42] documented that volunteering is associated with increased longevity, improved ability to carry out activities of daily living, better health coping mechanisms, adoption of healthy lifestyles, improved quality of life, social support, interaction and self-esteem. Other reviews that examined the impact of formally organised volunteering [2, 43] on volunteers’ physical and mental health, looked at the influence of the type of volunteering and its intensity on the health benefits observed. These cohort studies reported that volunteering has favourable effects on depression, life satisfaction and well-being, although not on physical health.

In the Phone Pal study, the desire for social distance of volunteers towards patients decreased, which could indicate a reduction of discriminatory attitudes. However, it must be noted that attitudes do not always predict or determine behaviour. It is possible to affect participants’ behaviour with a persuasive system even if their attitudes toward the behaviour are not favourable [44]. This is supported by the theory of cognitive consistency, which proposed that one can often proceed more efficiently from behaviour to attitudes [45]. If the behaviour changes first, for example by legal constraints, it may be expected that the attitude change will follow [45]. The opposite may not always happen. With respect to social comparison, the overall ratings of volunteers increased in all areas, except in the perception of their talent. This might not be surprising since, in this sample, volunteers had higher education attainment and employment conditions, and their self-concept could have improved following their one-to-one interactions with their matched patient.

Although differences in sub-scales ratings were observed in this feasibility study, these details are not expanded upon in this manuscript; further investigation of these findings should be conducted in future research with a larger sample. Importantly, some of the utilised measures had low alpha reliabilities on sub-scales and therefore data should be interpreted with caution. Although there were variations in these outcome measures in this within group comparison, it is inappropriate to provide any indication of impact. This is a small feasibility study not powered to test effectiveness, and the changes observed overall relate to participants at the beginning and end of the study. The findings from participants who received the intervention cannot be compared to a control group. Whilst it may be encouraging that several measures demonstrated improvement in the follow-up assessments, these apparent increased benefits could be explained by the Hawthorne effect [46], i.e. influenced by participating in a research study or by social desirability bias, leading to both patients and volunteers inflating their responses at follow-up. Still, the current quantitative and qualitative data together suggest that this intervention shows promise of success in the intended population.

## Conclusions

The findings of this feasibility study have demonstrated that connecting patients with psychosis to volunteers in the community through smartphones is feasible, acceptable and safe. Additionally, the study showed that participants found it acceptable to monitor their written communication, although in practice, this turned out not to be feasible. Only one patient and one volunteer were lost to follow up; no one withdrew after starting the intervention.

## References

[1] Hallett C., et al., Volunteering in the care of people with severe mental illness: a systematic review. BMC Psychiatry, 2012. 12: p. 226. 23237048 10.1186/1471-244X-12-226 PMC3534251

[2] Jenkinson C.E., et al., Is volunteering a public health intervention? A systematic review and meta-analysis of the health and survival of volunteers. BMC Public Health, 2013. 13(13, 773.): p. 773. doi: 10.1186/1471-2458-13-773 23968220 PMC3766013

[3] Priebe S., et al., Effectiveness of a volunteer befriending programme for patients with schizophrenia: randomised controlled trial. Br J Psychiatry, 2020. 217(3): p. 477–483. doi: 10.1192/bjp.2019.42 30829190 PMC7116000

[4] NHS England, *The Five Year Forward View For Mental Health*. *London*: *Mental Health Task Force*. 2016.

[5] Firth J., et al., Mobile Phone Ownership and Endorsement of "mHealth" Among People With Psychosis: A Meta-analysis of Cross-sectional Studies. Schizophr Bull, 2016. 42(2): p. 448–55. doi: 10.1093/schbul/sbv132 26400871 PMC4753601

[6] de Sousa P., et al., The role of social isolation and social cognition in thought disorder. Psychiatry Res, 2018. 269: p. 56–63. doi: 10.1016/j.psychres.2018.08.048 30145302

[7] Pinto da Costa M. and Phone Pal Advisory Groups, An Intervention to Connect Patients With Psychosis and Volunteers via Smartphone (the Phone Pal): Development Study. JMIR Form Res, 2022. 6(6): p. e35086. doi: 10.2196/35086 35653171 PMC9204578

[8] Craig P., et al., Developing and evaluating complex interventions: the new Medical Research Council guidance. BMJ, 2008. 337: p. a1655. doi: 10.1136/bmj.a1655 18824488 PMC2769032

[9] Yardley L., et al., The person-based approach to intervention development: application to digital health-related behavior change interventions. J Med Internet Res, 2015. 17(1): p. e30. doi: 10.2196/jmir.4055 25639757 PMC4327440

[10] Orsmond G.I. and Cohn E.S., The Distinctive Features of a Feasibility Study: Objectives and Guiding Questions. OTJR (Thorofare N J), 2015. 35(3): p. 169–77. doi: 10.1177/1539449215578649 26594739

[11] Pinto da Costa M. and Phone Pal Advisory Groups, Volunteering via Smart-Phone for People With Psychosis-Protocol of a Feasibility Trial. Front Psychiatry, 2021. 12: p. 742202. doi: 10.3389/fpsyt.2021.742202 34916970 PMC8669436

[12] Priebe S., et al., Application and results of the Manchester Short Assessment of Quality of Life (MANSA). Int J Soc Psychiatry, 1999. 45(1): p. 7–12. doi: 10.1177/002076409904500102 10443245

[13] Craig C.L., et al., International physical activity questionnaire: 12-country reliability and validity. Med Sci Sports Exerc, 2003. 35(8): p. 1381–95. doi: 10.1249/01.MSS.0000078924.61453.FB 12900694

[14] Lecomte T., Corbiere M., and Laisne F., Investigating self-esteem in individuals with schizophrenia: relevance of the Self-Esteem Rating Scale-Short Form. Psychiatry Res, 2006. 143(1): p. 99–108. doi: 10.1016/j.psychres.2005.08.019 16725210

[15] Allan S. and Gilbert P., A social comparison scale: Psychometric properties and relationship to psychopathology. Personality and Individual Differences, 1995. 19(3): p. 293–299.

[16] Collins N.L., Working models of attachment: Implications for explanation, emotion, and behavior. Journal of Personality and Social Psychology, 1996. 71(4): p. 810–832. doi: 10.1037//0022-3514.71.4.810 8888604

[17] Giacco D., et al., Social contacts and loneliness in people with psychotic and mood disorders. Compr Psychiatry, 2016. 66: p. 59–66. doi: 10.1016/j.comppsych.2015.12.008 26995237

[18] Ventura J., Green M. F., Shaner A., & Liberman R. P., Training and quality assurance with the Brief Psychiatric Rating Scale: "The drift busters". International Journal of Methods in Psychiatric Research, 1993. 3(4): p. 221–244.

[19] McGuire-Snieckus R., et al., A new scale to assess the therapeutic relationship in community mental health care: STAR. Psychol Med, 2007. 37(1): p. 85–95. doi: 10.1017/S0033291706009299 17094819

[20] Link B.G., et al., The Social Rejection of Former Mental Patients: Understanding Why Labels Matter. American Journal of Sociology, 1987. 92(6): p. 1461–1500.

[21] Shrive F.M., et al., Dealing with missing data in a multi-question depression scale: a comparison of imputation methods. BMC Med Res Methodol, 2006. 6: p. 57. doi: 10.1186/1471-2288-6-57 17166270 PMC1716168

[22] DG A., *Practical statistics for Medical Research*. London: Chapman & Hall/CRC., 1991

[23] Murray E., et al., Methodological challenges in online trials. J Med Internet Res, 2009. 11(2): p. e9. doi: 10.2196/jmir.1052 19403465 PMC2762798

[24] Pinto da Costa M., et al., How would patients with psychosis like to be in contact with a volunteer: Face-to-face or digitally? PLoS One, 2019. 14(5): p. e0216929. doi: 10.1371/journal.pone.0216929 31095611 PMC6522036

[25] Lauber C., et al., Determinants of attitude to volunteering in psychiatry: results of a public opinion survey in Switzerland. Int J Soc Psychiatry, 2002. 48(3): p. 209–19. doi: 10.1177/002076402128783253 12413249

[26] Eldridge S.M., et al., CONSORT 2010 statement: extension to randomised pilot and feasibility trials. Pilot Feasibility Stud, 2016. 2: p. 64. doi: 10.1186/s40814-016-0105-8 27965879 PMC5154046

[27] Boonstra T.W., et al., Using Mobile Phone Sensor Technology for Mental Health Research: Integrated Analysis to Identify Hidden Challenges and Potential Solutions. J Med Internet Res, 2018. 20(7): p. e10131. doi: 10.2196/10131 30061092 PMC6090171

[28] Orr K., et al., Validity of smartphone pedometer applications. BMC Res Notes, 2015. 8: p. 733. doi: 10.1186/s13104-015-1705-8 26621351 PMC4666074

[29] Locke, M., Ellis, A., Smith, J.D., ‘*Hold on to what you’ve got*: *the volunteer retention literature*’ Voluntary Action, 2003. 5((3)): p. 81–99.

[30] Yanay G.V. and Yanay N., The decline of motivation?: From commitment to dropping out of volunteering. Nonprofit Management and Leadership, 2008. 19(1): p. 65–78.

[31] Schulz K.F. and Grimes D.A., Sample size slippages in randomised trials: exclusions and the lost and wayward. The Lancet, 2002. 359(9308): p. 781–785. doi: 10.1016/S0140-6736(02)07882-0 11888606

[32] Miller B.J., et al., How connected are people with schizophrenia? Cell phone, computer, email, and social media use. Psychiatry Res, 2015. 225(3): p. 458–63. doi: 10.1016/j.psychres.2014.11.067 25563669

[33] Pinto da Costa M., Kouroupa A., and Virdi K., What is it like to communicate with a Phone Pal? The views and experiences of patients with psychosis and volunteers. SSM—Qualitative Research in Health, 2023. 3: p. 100221. doi: 10.1016/j.ssmqr.2023.100221

[34] Phillips R., et al., Analysis and reporting of adverse events in randomised controlled trials: a review. BMJ Open, 2019. 9(2): p. e024537. doi: 10.1136/bmjopen-2018-024537 30826796 PMC6398660

[35] Yang C.-c. and Robinson A., Not necessarily detrimental: Two social comparison orientations and their associations with social media use and college social adjustment. Computers in Human Behavior, 2018. 84: p. 49–57.

[36] Tison G.H. and Marcus G.M., Will the smartphone become a useful tool to promote physical activity? The Lancet Digital Health, 2019. 1(7): p. e322–e323. doi: 10.1016/S2589-7500(19)30154-2 33323203

[37] Zagalaz-Sanchez M.L., et al., Mini Review of the Use of the Mobile Phone and Its Repercussion in the Deficit of Physical Activity. Front Psychol, 2019. 10: p. 1307. doi: 10.3389/fpsyg.2019.01307 31244720 PMC6563677

[38] Shcherbina A., et al., The effect of digital physical activity interventions on daily step count: a randomised controlled crossover substudy of the MyHeart Counts Cardiovascular Health Study. The Lancet Digital Health, 2019. 1(7): p. e344–e352. doi: 10.1016/S2589-7500(19)30129-3 33323209

[39] Direito A., et al., mHealth Technologies to Influence Physical Activity and Sedentary Behaviors: Behavior Change Techniques, Systematic Review and Meta-Analysis of Randomized Controlled Trials. Ann Behav Med, 2017. 51(2): p. 226–239. doi: 10.1007/s12160-016-9846-0 27757789

[40] Umberson D. and Montez J.K., Social relationships and health: a flashpoint for health policy. J Health Soc Behav, 2010. 51 Suppl: p. S54–66. 20943583 10.1177/0022146510383501 PMC3150158

[41] Shevlin M., et al., Adult attachment styles and the psychological response to infant bereavement. Eur J Psychotraumatol, 2014. 5. doi: 10.3402/ejpt.v5.23295 24839541 PMC4023106

[42] Casiday R, K.E., Fisher C, Bambra C, *Volunteering and health*: *what impact does it really have*? *Final report to Volunteering England*, in *London*, *UK*: *Volunteering England*;. 2008. p.

[43] Ellis Paine A, H.M., Rochester C, *‘A rose by any other name…’ Revisiting the question*: *‘what exactly is volunteering*?*’ Working paper series*: *Paper 1*. *London*, *UK*: *Institute for Volunteering Research*. 2010.

[44] Oinas-Kukkonen, H. and M. Harjumaa, *Persuasive Systems Design*: *Key Issues*, *Process Model*, *and System Features*. Communications of the Association for Information Systems, 2009. 24.

[45] McGuire W.J., “Persuasion” *in* Miller G. A. (ed.) *Communication*, *Language*, *and Meaning Psychological Perspectives*, New York: Basic Books,. 1973: p. pp. 242–255.

[46] Franke R.H. and Kaul J.D., The Hawthorne Experiments: First Statistical Interpretation. American Sociological Review, 1978. 43(5): p. 623.

